# 2121. *In vitro* Synergy of the Combination of Sulbactam-Durlobactam and Cefepime at Clinically Relevant Concentrations Against *A. baumannii*, *P. aeruginosa*, and Enterobacterales

**DOI:** 10.1093/ofid/ofad500.1744

**Published:** 2023-11-27

**Authors:** Aliaa Fouad, David P Nicolau, Christian M Gill

**Affiliations:** Hartford Hospital, Farmington, Connecticut; Hartford Hospital, Farmington, Connecticut; Hartford Hospital, Farmington, Connecticut

## Abstract

**Background:**

Due to the incidence of polymicrobial nosocomial infections, effective therapies with activity against *Acinetobacter baumannii* (ACB), *P. aeruginosa* (PSA) and Enterobacterales are needed. Sulbactam-durlobactam (SUL-DUR) has potent, selective activity against ACB. Cefepime (FEP) is a common first-line therapy for hospital/ventilator associated pneumonia caused by non-ACB Gram-negative pathogens, but resistance to FEP is increasing. This study investigated the *in vitro* synergy of SUL-DUR + FEP against relevant pathogens.

**Methods:**

Static time-kills were performed in duplicate against fourteen FEP-resistant isolates (*ACB*, n=4; *PSA*, n=4; *E. coli, n=3*; *K. pneumoniae*, n=3). One wild type *K. pneumoniae* isolate was included. Antibiotic concentrations simulated the free-steady state average concentration of clinically administered doses in patients (SUL-DUR 1g-1g q6h, FEP 2g q8h).

**Results:**

SUL-DUR alone showed significant activity against ACB consistent with the MIC for isolates at 8 mg/L and lower (range: 0.91-2.14 log_10_ reduction in CFU/mL relative to 0h control). SUL-DUR plus FEP showed synergy (2.96 log_10_ reduction in CFU/mL relative to SUL-DUR alone) against one *ACB* isolate with an elevated MIC to SUL-DUR (32 mg/L). SUL-DUR plus FEP showed synergy vs. all PSA isolates (range: 2.93-3.27 log_10_ reduction in CFU/mL relative to SUL-DUR alone). Against Enterobacterales, combination therapy was indifferent due to significant kill from SUL-DUR (due to the intrinsic antibacterial activity of DUR alone vs. Enterobacterales) with ∼ 4 log_10_ kill relative to 0h. SUL-DUR plus FEP was synergistic (5.82 log_10_ reduction in CFU/mL relative to SUL-DUR alone) vs. one *E. coli* isolate with an elevated MIC to SUL-DUR ( >128 mg/L). SUL-DUR plus FEP showed synergy against one of the *K. pneumoniae* with 2.97 log_10_ CFU/mL reduction relative to SUL-DUR and an additive effect against the other two *K. pneumoniae* tested (range: 1.82-1.94 log_10_ reduction in CFU/mL relative to SUL-DUR alone). No antagonism was observed in any isolates.

**Conclusion:**

Synergy and no antagonism was observed with the combination of SUL-DUR and FEP. Further pharmacokinetic/pharmacodynamic studies conducted *in vivo* to verify these results are warranted.

**Disclosures:**

**David P. Nicolau, PharmD**, Allergan: Advisor/Consultant|Allergan: Grant/Research Support|Cepheid: Advisor/Consultant|Cepheid: Grant/Research Support|Merck: Advisor/Consultant|Merck: Grant/Research Support|Pfizer: Advisor/Consultant|Pfizer: Grant/Research Support|Shionogi: Advisor/Consultant|Shionogi: Grant/Research Support|Tetraphase: Advisor/Consultant|Tetraphase: Grant/Research Support|Venatorx: Advisor/Consultant|Venatorx: Grant/Research Support|Wockhardt: Advisor/Consultant|Wockhardt: Grant/Research Support **Christian M. Gill, PharmD**, Cepheid: Grant/Research Support|Entasis therapeutics: Grant/Research Support|Everest Medicines: Grant/Research Support|Shionogi: Grant/Research Support

